# Clinical Outcomes of the Canine Bypass Anchorage Technique for Severe Maxillary Bone Deficiency: A Case Report Series

**DOI:** 10.3390/reports9020195

**Published:** 2026-06-22

**Authors:** Calin Romulus Fodor, Marta Bieńkowska, Bartosz Dalewski, Łukasz Pałka

**Affiliations:** 1 Fodor Romulus Calin’s Clinic of Dentistry and Implantology, Str. Dragoş Vodă 8, 405300 Gherla, Romania; 2 REG-MED Dental Clinic, Rzeszowska 2, 68-200 Żary, Poland; 3 Department of Dental Prosthetics, Pomeranian Medical University, 70-204 Szczecin, Poland; bartosz.dalewski@pum.edu.pl; 4 Private Dental Practice, Rzeszowska 2, 68-200 Żary, Poland

**Keywords:** dental implants, cortical anchorage, immediate loading, maxillary atrophy, osseointegration

## Abstract

**Background/Objectives:** Advanced implant anchorage techniques are increasingly used to manage severe maxillary bone deficiency and to avoid extensive bone augmentation procedures. This case series report aimed to describe the canine bypass anchorage technique and to evaluate the short- to medium-term clinical outcomes and survival of implants placed using this approach. **Materials and Methods:** Thirteen patients presenting with missing maxillary premolars or posterior segments and insufficient alveolar bone height for conventional axial implant placement were treated using the canine bypass technique. A total of 19 long one-piece implants were inserted palatally to the canine root, engaging distant cortical bone of the nasal cavity and/or palatal alveolar process. Pre- and postoperative cone-beam computed tomography (CBCT) examinations were performed to assess implant positioning and anchorage. Patients were followed up to 3.5 years. **Results:** The mean follow-up period was 26.1 ± 10.8 months. Nasal cortical anchorage was achieved in 84.2% of implants, and palatal cortical anchorage in 73.7%; both anchorage types were obtained simultaneously in 57.9% of cases. The mean distance between the implant and canine root was 1.27 ± 1.4 mm (range: −1.0 to 4.5 mm), including cases of direct implant–tooth contact and periodontal ligament space transgression. All implants remained functional throughout the observation period, yielding a cumulative survival rate of 100%. Canine pulp vitality was preserved in all non-endodontically treated teeth. **Conclusions:** Within the limitations of this case series report, the canine bypass anchorage technique appears to be a feasible and minimally invasive treatment option for maxillary rehabilitation with implant-supported restoration in selected patients with severe bone deficiency, potentially allowing avoidance of sinus augmentation procedures. Further prospective studies with larger patient cohorts and longer follow-up periods are required to confirm the long-term safety, predictability, and clinical applicability of this approach.

## 1. Introduction

Advances in implant design, surgical instrumentation, and digital planning have expanded the possibilities for implant anchorage in patients with challenging anatomical conditions. Recently, new techniques have been introduced, such as transcrestal implants [1], extrasinus infratemporal anchorage [2], transnasal implants [3], and glabella implants [4]. These developments have been facilitated by advances in implant design, surgical guides, and digital planning technologies [5,6]. Consequently, advanced anchorage approaches are increasingly applied in patients presenting with severe alveolar atrophy, compromised bone quality, or limited implant sites. Initially, strategies to address these challenges were considered in full-arch implant-supported rehabilitations; however, they are now also increasingly relevant in partially dentate cases, including so-called segmental rehabilitation in the maxilla. Nevertheless, maintaining stable peri-implant bone conditions remains one of the key determinants of long-term implant success and depends on multiple biological and anatomical factors affecting bone preservation around the implant platform [7].

From the perspective of all dental specialties, the canine plays a crucial role. Beyond its importance in the esthetic zone, the canine contributes significantly to mastication, control of tooth wear, phonetics, and guided occlusion; canine guidance is widely regarded as a key functional component [8]. Preoperative evaluation of the sagittal root position (SRP) and surrounding bone morphology using cone-beam computed tomography (CBCT) is considered essential for safe implant planning in the anterior maxilla [9]. Moreover, the canine-associated bone protuberance forms a natural anatomical buttress and delineates the curvature of the dental arch, which often limits the available implantation area and may reduce the need for bone augmentation procedures such as sinus lift surgery. As a primary force-transmission pillar, the canine typically has the longest root, surrounded by well-mineralized bone, and is therefore often among the last teeth retained in elderly or periodontally compromised patients [10]. At the same time, canines frequently exhibit labially positioned roots and may present with bone fenestration or dehiscence [11,12].

The arch position and root morphology of canines allow the bone located posterior to the tooth to be utilized for implant placement. Considering both biological and financial costs, as well as current recommendations for implant-supported rehabilitation, preservation of the canine whenever possible appears clinically justified. In clinical reality, however, when a canine is replaced with an implant, canine guidance is usually superseded in favor of group function. This underscores the fact that a natural tooth, together with its periodontal apparatus and proprioceptive receptors, is functionally more valuable than the mere availability of bone for implant placement in this region [13,14]. To date, the term “canine bypass implant” has most commonly been associated with impacted teeth. In clinical scenarios where an impacted canine obstructs access to alveolar bone, the clinician may choose to drill through the tooth, extract it, or bypass it by placing the implant from either the buccal or palatal aspect.

Clinical challenges arise when the distance between the canine and posterior teeth is too large to allow safe prosthetic span lengths, or when insufficient bone volume is present to place an implant distal to the canine [15,16,17,18]. To address these limitations, the canine bypass technique was introduced by A.Lazarov [19].

The technique proposed in this study describes a situation in which implant bypasses the canine from the palatal side, with its apex engaging one of the nasal cavity walls to achieve stable cortical anchorage. This approach utilizes long one-piece implants capable of engaging distant cortical structures, thereby expanding the range of treatment options available in selected patients with severe maxillary bone deficiency. The objectives of this case series report were to describe the canine bypass anchorage technique and its surgical protocol, evaluate the radiographic relationship between the implant and the adjacent canine root and to assess implant survival and canine vitality during functional loading with a follow-up period of up to 3.5 years.

## 2. Material and Methods

**Figure 1:**
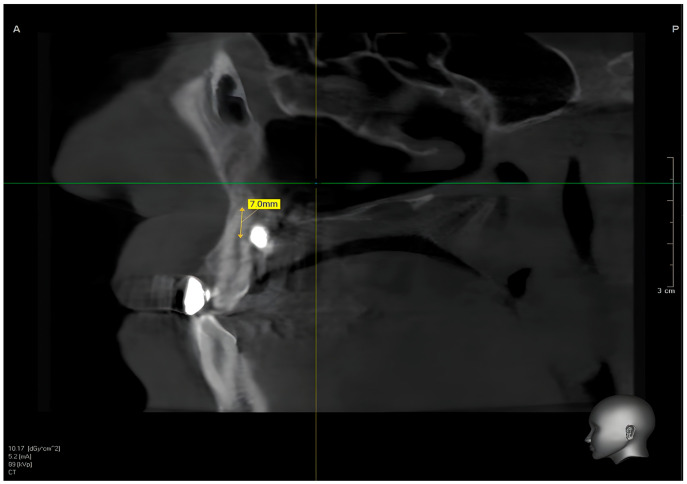
Postoperative sagittal CBCT image showing the spatial relationship between the canine root and the dental implant. The measured distance between the structures is indicated (7.0 mm), illustrating the achieved anchorage and confirming safe positioning of the implant relative to the adjacent tooth root.

**Figure 2:**
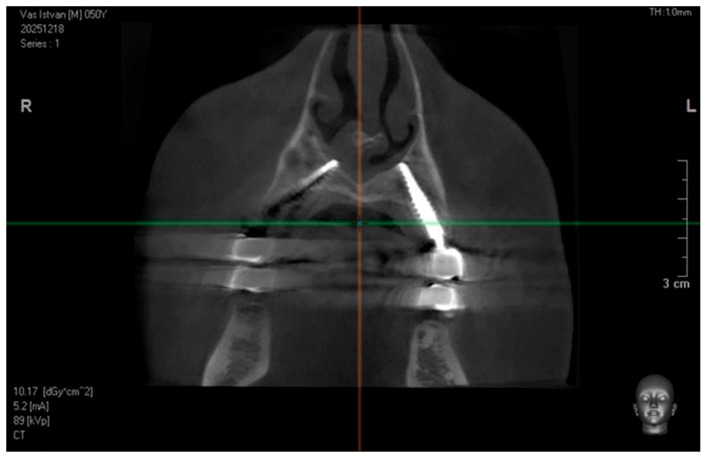
Postoperative coronal CBCT image demonstrating bilateral implant positioning and the achieved anchorage pattern, confirming stable placement and appropriate angulation in relation to surrounding anatomical structures.

This case series report included 13 patients presenting with missing maxillary premolars or posterior segments (premolars and molars) on at least one side of the maxilla, combined with insufficient alveolar bone height to support axially placed dental implants in the premolar region. All patients underwent implant placement using the canine bypass technique. Preoperative cone-beam computed tomography (CBCT) was used to assess bone conditions and the status of the canines. Postoperative CBCT scans and follow-up examinations were performed to confirm the relationship between the canine root and the implant, as well as the type of anchorage achieved (Figure 1 and Figure 2).

Inclusion criteria were missing maxillary premolars and molars; insufficient alveolar bone high for conventional implant placement in the posterior region; presence of a natural canine adjacent to the edentulous area; and availability of preoperative and postoperative CBCT examinations with a minimum follow-up period of 12 months.

Exclusion criteria included active periodontal disease affecting the adjacent canine, uncontrolled systemic conditions potentially compromising osseointegration, inadequate radiographic documentation, and follow-up shorter than 12 months. Patients treated between January 2022 and July 2025 were included in the study. No formal sample size calculation was performed because of the retrospective observational design. All eligible patients treated during the study period were included. Implant-supported restorations were delivered three days after surgery. Occlusal contacts were adjusted to minimize excessive lateral loading during the healing phase. Postoperative management included antibiotic prophylaxis, non-steroidal anti-inflammatory medication as required, and chlorhexidine mouth rinses for 14 days. The study was conducted in accordance with the Declaration of Helsinki and was approved by the Chairman of the Bioethics Committee at the District Medical Chamber (Decision No. 03/179/2025 ZG).

### 2.1. Surgical Protocol

After mucosal disinfection with a povidone–iodine solution (Betadine^®^, Avrio Health L.P., Stamford, CT, USA) and local administration of anesthesia using 4% articaine with epinephrine 1:100,000 (Ubistesin Forte^®^, Pierrel S.p.A., Capua, Italy), the procedure was performed either flapless or using a full-thickness mucoperiosteal flap. A flapless approach was preferred when sufficient keratinized mucosa and favorable anatomical conditions were present during clinical and on preoperative CBCT examination. A full-thickness mucoperiosteal flap was elevated when direct visualization of the alveolar ridge morphology or anatomical structures was considered necessary. A 1:1 handpiece operating at 20,000 rpm with external saline irrigation was used throughout the osteotomy preparation.

**Figure 3:**
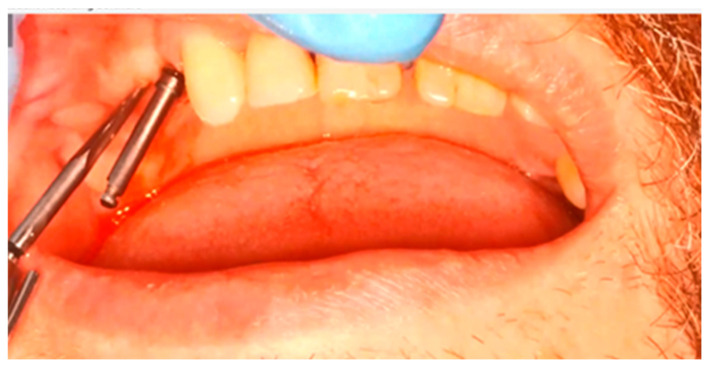
Intraoperative view of the osteotomy procedure showing the use of two sequential drills: the pilot (pathfinder) drill (BCDX1) followed by a 2.0 mm diameter, 40 mm long twist drill. The initial drilling point was located at the center of the alveolar ridge in the first premolar region, with the drilling trajectory oriented at approximately 45° to the ridge plane.

Two drills were required for the osteotomy procedure: a pilot (pathfinder) drill (BCDX1), followed by a 2.0 mm diameter, 40 mm long twist drill (Figure 3). The initial drilling point was located at the center of the alveolar ridge in the first premolar region, with an angulation of approximately 45° relative to the ridge plane. In the bucco-palatal direction, the drilling trajectory was guided by intraoral tactile control using the thumb–index technique to identify the trabecular pathway between the cortical plate of the palatine process and the canine root, situated within the trabecular bone between the cortical plate of the palatine process of the maxilla and the canine root.

Osteotomy preparation was continued until the nasal cortical plate was reached. The osteotomy depth was determined using laser markings on the drill, after which the cortical bone was perforated to achieve stable cortical anchorage. In cases where a double bypass was required—most commonly due to compromised sinus conditions—a second osteotomy was prepared parallel to the first, originating in the region of the second premolar. A double bypass approach was selected when a single implant was considered insufficient to provide the desired prosthetic support or when sinus anatomy limited the availability of distal anchorage sites. Implant placement was performed using one-piece cortical implants (IHDE Dental, Switzerland) with diameters ranging from 3.5 to 5.5 mm and lengths ranging from 15 to 29 mm.

**Figure 4:**
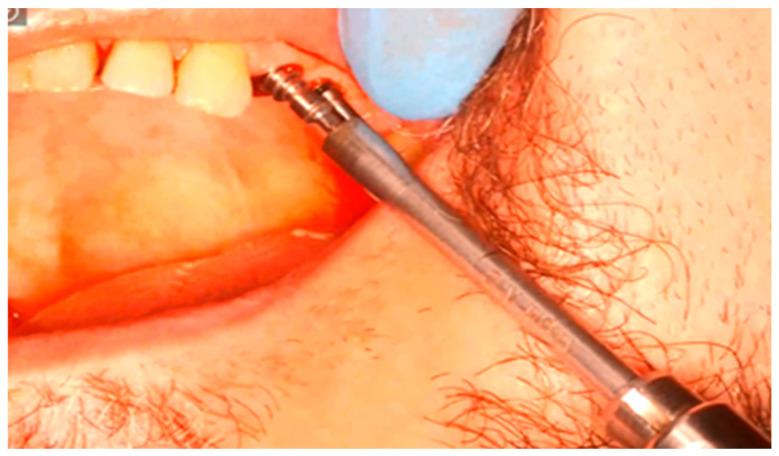
Intraoperative view of implant placement following completion of the osteotomy. The implant is inserted using a hand-grip insertion tool, with final insertion torque exceeding 50 N·cm, indicating adequate primary stability and effective cortical anchorage.

After completion of the osteotomy, the selected implant was inserted using a hand-grip insertion tool (Figure 4). Primary stability was assessed by insertion torque, which exceeded 50 N·cm in all successfully anchored implants.

### 2.2. Postoperative Management

Patients received postoperative instructions regarding oral hygiene and diet. Antimicrobial mouth rinses and analgesic medication were prescribed according to the standard protocol of the treating center.

### 2.3. Outcome Assessment

Implant survival was defined as the absence of implant mobility, infection, implant removal, or loss of prosthetic function during follow-up.

Canine vitality was assessed during follow-up using a cold test (Endo cold spray, Henry Schein, Melville, NY, USA). Teeth were considered vital when a positive response was obtained in at least one test spot and no clinical or radiographic signs of pulp pathology were present.

Radiographic assessment was performed using cone-beam computed tomography (CBCT) scans acquired with a Pax-i3D Smart Tomograph (Vatech Co., Ltd., Hwaseong, Republic of Korea). Measurements of the shortest distance between the implant surface and the canine root were obtained on postoperative CBCT scans using Ez3D-i software version 1.0.6.0.1 (Vatech Co., Ltd., Hwaseong, Republic of Korea).Measurements were performed in sagittal and cross-sectional planes corresponding to the closest implant–root relationship. All measurements were performed by a single experienced examiner.

### 2.4. Statistical Analysis

Data were analyzed using Python version 3.12 (Python Software Foundation, Wilmington, DE, USA) with the Pandas library version 2.3.0. Descriptive statistics were used to summarize the study variables. Continuous data are presented as mean ± standard deviation (SD) or median (range), as appropriate. Given the small sample size and descriptive design of the study, no statistical hypothesis testing was performed.

## 3. Results

Thirteen patients received a total of 19 canine bypass implants. The observation period extended up to 3.5 years, with a mean follow-up duration of 26.1 ± 10.8 months. The study population consisted of nine men (69.2%) and four women (30.8%), with a mean age of 46.9 ± 10.96 years (range: 34–71 years). Within the study group, one patient was a smoker (10 cigarettes per day), one patient was diagnosed with hypertension and treated with nebivolol, and one patient had arrhythmia treated with flecainide. Detailed demographic, clinical, and implant characteristics are presented in Table 1.

**Figure 5:**
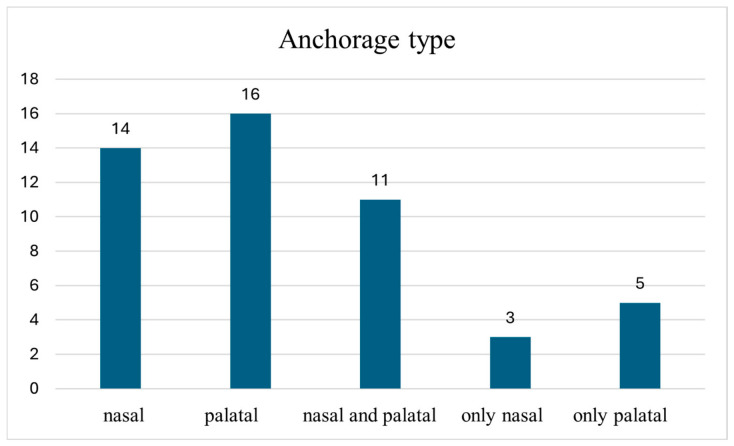
Types of cortical anchorage obtained.

The mean bypass distance between the implant and the canine root was 1.27 ± 1.4 mm, with a median value of 1.10 mm. The distance between the implant and canine root ranged from −1.0 mm to 4.5 mm (mean 1.27 ± 1.4 mm; median 1.10 mm). Distances greater than 2 mm were observed in five implants, distances between 1 and 2 mm in five implants, distances between 0 and 1 mm in three implants, direct implant–root contact in five implants, and periodontal ligament space transgression in one implant. Nasal cortical anchorage was achieved in 16 of 19 implants (84.2%), palatal cortical anchorage in 14 of 19 implants (73.7%), and combined anchorage in 11 implants (57.9%) (Figure 5).

Prior to implantation, 15 canines exhibited vital pulp, whereas four canines had undergone endodontic treatment. All non-endodontically treated canines maintained positive vitality responses throughout follow-up. The study population demonstrated a slight male predominance (male-to-female ratio = 2.25:1), with men comprising 69.2% of the cohort and women 30.8%.

### Implant Survival

During the observation period, no implant mobility, implant loss, biological complications requiring implant removal, were recorded. Consequently, the cumulative implant survival rate was 100%.

No intraoperative complications, postoperative infections or adverse events involving adjacent canines were observed during the follow-up period.

## 4. Discussion

The present study describes the canine bypass anchorage technique as a treatment option for patients with severe maxillary bone deficiency in whom conventional implant placement distal to the canine is not feasible. A total of 19 implants were placed in 13 patients, resulting in a cumulative survival rate of 100% during a mean follow-up period of 26.1 months. Preservation of canine vitality was observed in all non-endodontically treated teeth, including cases involving direct implant–tooth contact or periodontal ligament transgression.

The use of the palatine process of the maxilla or the cortical bone surrounding the nasal cavity for implant anchorage has been widely described in the literature [20,21]. These approaches are most applied in full-arch implant-supported rehabilitations, where no teeth, or at least no canines, are present during osteotomy preparation, thus allowing unobstructed access to distant anterior cortical bone. In situations where canines are present in the maxilla, clinical management is typically limited to either extraction of the tooth or its incorporation into the prosthetic restoration. These approaches allow the clinician to utilize distant cortical bone to achieve high primary stability while avoiding extensive augmentation procedures.

Under favorable anatomical conditions, such as Class I according to the Kan classification [12], where sufficient bone volume is present on the palatal aspect of the canine, it is possible to bypass the canine with an implant and engage the distant cortical bone of the anterior maxilla. CBCT-based studies have demonstrated that Class I sagittal root position is the most common configuration in the anterior maxilla and provides favorable conditions for implant placement due to the availability of palatal bone for implant anchorage [9].

The described approach, referred to as the canine bypass technique, was first introduced in clinical practice in 2014. Anitua et al. reported a survival rate of 97.64% for implants utilizing nasal cavity cortical bone for anchorage over a 2.5-year observation period [22]. Similarly, Peñarrocha-Oltra et al. reported a 5-year success rate of 98.7% for palatally placed implants [23].

In the present study, 11 implants achieved anchorage in the nasal cortical bone, and in 12 implants, the cortical bone of the palatine alveolar process was used for primary or additional stabilization, resulting in a 100% survival rate during the follow-up period. Comparable results were reported by Peñarrocha et al., who observed a 97.9% implant survival rate 12 months after loading when utilizing the anterior maxillary buttress for implant anchorage [21]. Although the present series demonstrated a 100% implant survival rate, this finding should be interpreted cautiously given the limited sample size and observational design. Nevertheless, the observed outcomes are broadly consistent with previously published reports describing distant cortical anchorage in the anterior maxilla.

According to the literature, a minimum distance of 1.5–2.0 mm between a dental implant and the root of an adjacent natural tooth is recommended to ensure clinical success [24,25]. Previous studies have also evaluated the influence of implant placement on the hard and soft tissues of adjacent teeth and generally reported favorable biological responses when appropriate surgical planning is performed [26]. In contrast, in the present series, this distance ranged from −1.0 mm to 4.5 mm without negatively affecting implant or tooth survival. This finding is in agreement with the results reported by Yi et al., who observed a 96.9% implant survival rate, with 90.6% of injured adjacent teeth remaining functional [27]. Recently, based on a systematic literature review, Labidi et al. proposed a paradigm shift, presenting successful implant placement in contact with dental tissues, particularly in cases involving impacted teeth [28]. The findings of the present study are consistent with emerging evidence suggesting that implant proximity to dental tissues does not invariably result in loss of tooth vitality or implant failure. However, the available evidence remains controversial as Han et al. reported that implant–tooth proximity and direct implant–root contact may be associated with adverse outcomes, including loss of pulp vitality and peri-implantitis, and emphasized that such situations should generally be regarded as iatrogenic events requiring prevention whenever possible [29]. Furthermore, recent large-scale cohort data have suggested that teeth adjacent to dental implants may be at increased risk of long-term complications, particularly root fractures [30]. Therefore, although no biological complications were observed in the present series, the long-term safety of implant–root contact remains uncertain and should be interpreted with caution.

The preservation of natural maxillary canines remains an important therapeutic objective whenever feasible. Beyond their functional role in occlusal guidance and force distribution, canines contribute significantly to the maintenance of periodontal and esthetic stability. A recent systematic review evaluating periodontal outcomes of surgically–orthodontically treated impacted maxillary canines reported generally favorable long-term periodontal conditions, further supporting treatment strategies aimed at preserving natural canines whenever anatomically and functionally possible [31].

From a clinical perspective, the canine bypass technique may be particularly useful in patients presenting with severe posterior maxillary atrophy, insufficient bone volume distal to the canine for conventional implant placement, and a desire to avoid sinus augmentation procedures while preserving a strategically important natural canine. The technique appears most suitable when adequate palatal bone volume is available and favorable canine root morphology allows safe implant passage toward distant cortical anchorage. Conversely, unfavorable sagittal root position, insufficient palatal bone, active periodontal disease affecting the adjacent canine, or the inability to achieve stable cortical fixation may limit the applicability of this approach. Therefore, careful CBCT-based treatment planning and appropriate case selection remain essential prerequisites for successful implementation of the canine bypass concept.

In the current series, direct contact between the implant and the canine occurred in three patients, and in one case, the implant transgressed the periodontal space. Among these cases, three canines had not undergone endodontic treatment and maintained pulp vitality throughout the observation period. Direct implant–root contact was not the primary surgical objective but occurred in a limited number of cases due to anatomical constraints and the need to obtain stable cortical anchorage. No radiographic signs of root resorption, periapical pathology, or loss of tooth vitality were observed during the available follow-up period.

From a biomechanical perspective, engagement of distant cortical structures may increase primary stability and facilitate immediate loading. Cortical anchorage distributes functional forces differently from conventional trabecular anchorage and may be advantageous in severely resorbed maxillae where bone quantity and quality are limited. Adequate primary stability and control of implant micromotion are widely recognized as key prerequisites for successful osseointegration, particularly when immediate loading protocols are employed. Furthermore, recent evidence suggests that insertion torque and implant stability represent related but distinct biomechanical parameters, highlighting the complex relationship between mechanical anchorage and biological response [32,33,34]. Recent systematic evidence has also demonstrated that immediate and early loading protocols can achieve high survival rates in partially edentulous patients when appropriate case selection and sufficient primary stability are achieved [35]. However, the long-term biomechanical behavior of canine bypass implants requires further investigation.

Compared with sinus augmentation procedures, the canine bypass approach may reduce treatment time, morbidity, and the need for additional grafting materials. However, contemporary graft-based regenerative techniques still remain optional treatment for patients with severe alveolar bone deficiency [36].

### Limitations

The present study has several limitations. First, the sample size was limited, and no control group was available for comparison. Second, all procedures were performed in a single center, potentially limiting generalizability. Third, the retrospective design prevented standardized collection of several clinically relevant parameters, including marginal bone loss, peri-implant soft tissue indices, patient-reported outcome measures, and quality-of-life assessments. Finally, the mean follow-up period of 26.1 months is insufficient to evaluate long-term biological and prosthetic outcomes, particularly in cases involving direct implant–root contact. Furthermore, all procedures were performed by clinicians experienced in cortical anchorage techniques, which may limit the reproducibility of the findings in less experienced clinical settings.

## 5. Conclusions

The canine bypass implant technique, when used alone or in combination with molar or tubero-pterygoid implants, can facilitate the surgical rehabilitation of patients presenting with severe maxillary resorption while avoiding sinus lift procedures. Nasal or palatal cortical fixation achieved through the canine bypass bone slot technique may represent a promising treatment option for maxillary rehabilitation in patients with limited bone availability, while potentially reducing the need for grafting procedures in selected cases. Successful application of this approach requires advanced surgical training and meticulous CBCT-based planning. Further prospective studies involving larger patient cohorts, longer follow-up periods, and standardized clinical outcome assessment are required to confirm the long-term safety, predictability, and clinical applicability of this approach.

## Figures and Tables

**Table 1:** Demographic, clinical, and implant characteristics of the study population.

Variable	Value
Number of patients	13
Number of implants	19
Mean age (years)	46.9 ± 10.96
Age range (years)	34–71
Male sex, n (%)	9 (69.2%)
Female sex, n (%)	4 (30.8%)
Mean follow-up (months)	26.1 ± 10.8
Maximum follow-up (years)	3.5
Smokers, n (%)	1 (7.7%)
Hypertension, n (%)	1 (7.7%)
Cardiac arrhythmia, n (%)	1 (7.7%)
Vital canines before implantation, n (%)	15
Endodontically treated canines, n (%)	4
Nasal cortical anchorage, n (%)	16 (84.2%)
Palatal cortical anchorage, n (%)	14 (73.7%)
Combined nasal and palatal anchorage, n (%)	11 (57.9%)
Implant survival rate, n (%)	19/19 (100%)
Preserved vitality of non-endodontically treated canines, n (%)	12/12 (100%)
Mean implant–canine distance (mm)	1.27 ± 1.4
Median implant–canine distance (mm)	1.10
Implant–canine distance range (mm)	−1.0 to 4.5

## Data Availability

The datasets generated and analyzed during the current study are available from the corresponding author on reasonable request.
